# Supplementary material for: Exploring Factors Associated with Physical Activity in the Elderly: A Cross-Sectional Study during the COVID-19 Pandemic
Source: Behav Sci (Basel). 2024 Jan 17;14(1):62. doi: 10.3390/bs14010062 (PMC10813266; doi:10.3390/bs14010062)
Supplement: Supplementary file 1 [file behavsci-14-00062-s001.zip › Supplementary table 1.pdf]

Supplementary table 1. Translated (Croatian) version of the PASE questionnaire

REDNI BROJ	AKTIVNOSTI SLOBODNOG VREMENA	ODGOVORI			
1	Tijekom proteklih 7 dana, koliko često ste provodili sjedilačke aktivnosti kao što je čitanje, gledanje TV-a ili ručni rad?	NISAM UOPĆE (prijeđite na pitanje 2)	RIJETKO (1-2 DANA) (prijeđite na pitanje 1a i 1b)	PONEKAD (1-2 DANA) (prijeđite na pitanje 1a i 1b)	ČESTO (1-2 DANA) (prijeđite na pitanje 1a i 1b)
1a	Koje su to bile aktivnosti?	upišite odgovor			
1b	U prosjeku koliko ste sati proveli u tim sjedilačkim aktivnostima?	< 1 sata	1-2 sata	>2-4 sata	> 4 sata
2	Tijekom proteklih 7 dana, koliko često ste hodali izvan doma ili dvorišta zbog bilo kojeg razloga? Primjerice, radi zabave ili vježbanja, šetnja do posla, šetanje psa, itd.?	NISAM UOPĆE (prijeđite na pitanje 3)	RIJETKO (1-2 DANA) (prijeđite na pitanje 2a i 2b)	PONEKAD (1-2 DANA)(prijeđite na pitanje 2a i 2b)	ČESTO (1-2 DANA)(prijeđite na pitanje 2a i 2b)
2a	U prosjeku koliko ste sati dnevno hodali?	< 1 sata	1-2 sata	>2-4 sata	> 4 sata
3	Tijekom proteklih 7 dana, koliko često ste sudjelovali u laganim sportskim ili rekreativnim aktivnostima kao što su kuglanje, balote (buće), pecanje iz barke i sl.?	NISAM UOPĆE (prijeđite na pitanje 4)	RIJETKO (1-2 DANA) (prijeđite na pitanje 3a i 3b)	PONEKAD (1-2 DANA) (prijeđite na pitanje 3a i 3b)	ČESTO (1-2 DANA) (prijeđite na pitanje 3a i 3b)
3a	Koje su to bile aktivnosti?	upišite odgovor			
3b	U prosjeku koliko ste sati proveli u tim laganim sportskim i rekreativnim aktivnostima?	< 1 sata	1-2 sata	>2-4 sata	> 4 sata

4	Tijekom proteklih 7 dana, koliko često ste sudjelovali u umjerenim sportskim i kreativnim aktivnostima kao što su tenis parovi, ples, lov, golf, stolni tenis i sl.?	NISAM UOPĆE (prijeđite na pitanje 5)	RIJETKO (1-2 DANA) (prijeđite na pitanje 4a i 4b)	PONEKAD (1-2 DANA) (prijeđite na pitanje 4a i 4b)	ČESTO (1-2 DANA) (prijeđite na pitanje 4a i 4b)
4a	Koje su to bile aktivnosti?	upišite odgovor			
4b	U prosjeku koliko ste sati proveli u tim umjerenim sportskim i rekreativnim aktivnostima?	< 1 sata	1-2 sata	>2-4 sata	> 4 sata
5	Tijekom proteklih 7 dana, koliko često ste provodili intenzivne/naporne sportske i rekreativne aktivnosti kao što su trčanje, plivanje, vožnja bicikla, tenis, aerobika, mali nogomet, skijanje i sl.	NISAM UOPĆE (prijeđite na pitanje 6)	RIJETKO (1-2 DANA) (prijeđite na pitanje 5a i 5b)	PONEKAD (1-2 DANA) (prijeđite na pitanje 5a i 5b)	ČESTO (1-2 DANA) (prijeđite na pitanje 5a i 5b)
5a	Koje su to bile aktivnosti?	upišite odgovor			
5b	U prosjeku koliko ste sati proveli u tim intenzivnim/napornim sportskim i rekreativnim aktivnostima?	< 1 sata	1-2 sata	>2-4 sata	> 4 sata
6	Tijekom proteklih 7 dana, koliko često ste učestvovali u bilo kakvoj vrsti specifičnog vježbanja radi poboljšanja snage i izdržljivosti, kao što su vježbe s utezima i	NISAM UOPĆE (prijeđite na pitanje 7)	RIJETKO (1-2 DANA) (prijeđite na pitanje 6a i 6b)	PONEKAD (1-2 DANA)(prijeđite na pitanje 6a i 6b)	ČESTO (1-2 DANA) (prijeđite na pitanje 6a i 6b)

	drugim opterećenjima (elastične trake, sprave za vježbanje, itd.)?				
6a	Koje su to bile aktivnosti?	upišite odgovor			
6b	U prosjeku koliko ste sati proveli u takvim sportskim i rekreativnim aktivnostima?	< 1 sata	1-2 sata	>2-4 sata	> 4 sata
**REDNI BROJ**	**KUĆNI POSLOVI/AKTIVNOSTI**				
7	Tijekom proteklih 7 dana, jeste li obavljali lagane kućanske poslove, kao što su brisanje prašine ili pranje posuđa?	Ne	Da		
8	Tijekom proteklih 7 dana, jeste li obavljali teške kućanske poslove ili zadatke, kao što su usisavanje, ribanje podova, pranje prozora ili prenošenje drva za ogrjev?	Ne	Da		
9	Tijekom proteklih 7 dana, jeste li obavljali neku od navedenih aktivnosti? Molimo odgovorite DA ili NE za svaku od aktivnosti.	Kućni popravci, bojanje, lijepljenje tapeta, električarski poslovi, itd.?	Košenje vrta i orezivanje biljaka okućnice, čišćenje snijega ili lišća, cijepanje drva, itd.?	Vrtlarenje na otvorenom	Briga o drugim osobama, primjerice djeca, bračni drug ili druge odrasle osobe?
**REDNI BROJ**	**RADNE AKTIVNOSTI**	**ODGOVORI**			
10	Tijekom proteklih 7 dana, jeste li radili za novac ili kao volonter?	Ne	Da (prijeđite na pitanje 10a i 10b)		

10a	Koliko sati tjedno ste radili za novac ili kao volonter?	Sati: _____			
10b	Koja od ovih kategorija najbolje opisuje količinu tjelesne aktivnosti koju zahtjeva taj posao/volontiranje koje ste obavljali?	Uglavnom sjedenje sa malim kretanjem ruku (uredski poslovi, nadzor, sjedeći poslovi slaganja, vožnja autobusa, itd.)	Sjedenje ili stajanje uz manje hodanje (blagajnik, upravljanje laganim alatima ili strojevima, itd.)	Hodanje uz određeno upravljanje materijalima lakšim od 25 kg (poštarski i dostavljački poslovi, konobarenje, rad na građevini, upravljanje teškim strojevima i alatima, itd.)	Hodanje i teški manualni rad koji često zahtjeva upravljanje materijalima težim od 25 kg (poslovi drvosječe, klesarski poslovi, fizički rad, itd.)
