# Supplementary material for: Exploring Factors Associated with Physical Activity in the Elderly: A Cross-Sectional Study during the COVID-19 Pandemic
Source: Behav Sci (Basel). 2024 Jan 17;14(1):62. doi: 10.3390/bs14010062 (PMC10813266; doi:10.3390/bs14010062)
Supplement: Supplementary file 1 [file behavsci-14-00062-s001.zip › Supplementary table 2.pdf]

Supplementary table 2. Test – retest percentage of equally responded queries for Physical Activity Scale for Elderly (PASE)

**LEISURE TIME PHYSICAL ACTIVITY**	**Test retest agreement**
Over the past 7 days, how often did you participate in sitting activities such as reading, watching TV, or doing handcrafts?	70%
What were these activities? (open end question)	45%
In average, how many hours did you engage in these sitting activities?	65%
Over the past 7 days, how often did you take a walk outside your home or yard for any reason? For example, for fun or exercise, walking to work, walking the dog, etc	60%
On average, how many hours per day did you spend walking?	54%
Over the past 7 days, how often did you engage in light sport or recreational activities such as bowling, golf with a cart, shuffleboard, fishing from a boat or pier or other similar activities?	69%
What were these activities?	42%
On average, how many hours did you engage in these light sport or recreational activities?	55%
Over the past 7 days, how often did you engage in moderate sport and recreational activities such as doubles tennis, ballroom dancing, hunting, ice skating, golf without a cart, softball or other similar activities?	59%
What were these activities?	41%
On average, how many hours did you engage in these moderate sport or recreational activities?	58%
Over the past 7 days, how often did you engage in strenuous sport and recreational activities such as jogging, swimming, cycling, singles tennis, aerobic dance, skiing (downhill or cross-country) or other similar activities?	63%
What were these activities?	46%
On average, how many hours did you engage in these strenuous sport or recreational activities?	59%
Over the past 7 days, how often did you do any exercises specifically to increase muscle strength and endurance, such as lifting weights or pushups, etc.?	69%
What were these activities?	60%
On average, how many hours did you engage in these strenuous sport or recreational activities?	70%
HOUSEHOLD ACTIVITY	
During the past 7 days, have you done any light housework, such as dusting or washing dishes?	75%
During the past 7 days, have you done any heavy housework or chores, such as vacuuming, scrubbing floors, washing windows, or carrying wood?	76%

During the past 7 days, did you engage in any of the following activities? Please answer YES or NO for each item.	69%
WORK RELATED ACTIVITY	
During the past 7 days, did you work for pay or as a volunteer?	60%
How many hours per week did you work for pay and or as a volunteer? ____ hours	41%
Which of the following categories best describes the amount of physical activity required on your job and or volunteer work?	72%
